# Bibliometric analysis of nutrition and dietetics research activity in Arab countries using ISI Web of Science database

**DOI:** 10.1186/2193-1801-3-718

**Published:** 2014-12-10

**Authors:** Waleed M Sweileh, Samah W Al-Jabi, Ansam F Sawalha, Sa’ed H Zyoud

**Affiliations:** Department of Pharmacology/Toxicology, College of Medicine and Health Sciences, An-Najah National University, Nablus, Palestine; Department of Clinical and Community Pharmacy, College of Medicine and Health Sciences, An-Najah National University, Nablus, Palestine

**Keywords:** Arab world, Nutrition, Dietetics, Bibliometric analysis, ISI, Web of Science

## Abstract

Reducing nutrition-related health problems in Arab countries requires an understanding of the performance of Arab countries in the field of nutrition and dietetics research. Assessment of research activity from a particular country or region could be achieved through bibliometric analysis. This study was carried out to investigate research activity in “nutrition and dietetics” in Arab countries. Original and review articles published from Arab countries in “nutrition and dietetics” Web of Science category up until 2012 were retrieved and analyzed using the ISI Web of Science database. The total number of documents published in “nutrition and dietetics” category from Arab countries was 2062. This constitutes 1% of worldwide research activity in the field. Annual research productivity showed a significant increase after 2005. Approximately 60% of published documents originated from three Arab countries, particularly Egypt, Kingdom of Saudi Arabia, and Tunisia. However, Kuwait has the highest research productivity per million inhabitants. Main research areas of published documents were in “Food Science/Technology” and “Chemistry” which constituted 75% of published documents compared with 25% for worldwide documents in nutrition and dietetics. A total of 329 (15.96%) nutrition - related diabetes or obesity or cancer documents were published from Arab countries compared with 21% for worldwide published documents. Interest in nutrition and dietetics research is relatively recent in Arab countries. Focus of nutrition research is mainly toward food technology and chemistry with lesser activity toward nutrition-related health research. International cooperation in nutrition research will definitely help Arab researchers in implementing nutrition research that will lead to better national policies regarding nutrition.

**Electronic supplementary material:**

The online version of this article (doi:10.1186/2193-1801-3-718) contains supplementary material, which is available to authorized users.

## Introduction

According to the World Health Organization, micronutrient deficiencies and underweight problems are common in the Eastern Mediterranean Region (EMR); (WHO/EMRO 2012). On the other hand, overweight and obesity have reached alarming limits in most of the countries in EMR (Musaiger 2011). Furthermore, prevalent health conditions in Arab countries such as diabetes mellitus, osteoporosis, and cardiovascular diseases have been linked to nutritional habits in the EMR (Musaiger 2011). Reducing nutrition-related health problems in Arab countries requires an understanding of the performance of Arab countries in the field of nutrition and dietetics research. Assessment of research activity from a particular country or region could be achieved through bibliometric analysis (Bramness et al. 2014; Zyoud et al. 2014c). Bibliometrics Analysis of research output from a particular country is an image of its research activity and its current economic, developmental and health status (De Battisti and Salini 2013). No bibliometric studies have carried out to assess research activity in the field of nutrition and dietetics from any Arab country or Arab region. Internationally, few studies in this regard were published. For example, a bibliometric analysis of nutrition literature of Bangladesh showed that a total of 636 articles were published in 100 local and foreign journals with a rapid growth of nutrition literature from 1987 onwards (Ahmed and Rahman 2008). Another study from China showed that the number of Chinese literature on clinical nutrition had increased steadily from 1 paper in 1974 to 1980 papers in 2011 and that clinical nutrition has become one of the hot research topics in China in recent years, although the appropriate and reasonable use of clinical nutrition remain challenging (Yu et al. 2013) Of course, research activity and capacity of a particular country depends on several factors including the national income and the size of population. In the case of Arab countries with approximately 400 million population and huge resources, excellence in research is a must. According to Arab League, there are 22 Arab countries and territories with varying degrees of income, population size, food industry and agricultural capacities. Arab countries, with approximately 400 million people, share similar language, culture, religion and historical background. Living standards, medical education, health services, political reform and awareness have become evident in most Arab countries. This should have been accompanied by increase in scientific research output. Therefore, the objective of this study was to analyze the research activity in nutrition and dietetics in Arab countries using bibliometric approach. The results of the study will provide baseline data on the quantity and quality of research in nutrition and dietetics category and will help direct research in this field in the future.

## Methodology

Data used in this study were extracted from ISI Web of Science (WoS) in which various scientific disciplines are being grouped into various categories based on the field of interest of journals indexed in ISI WoS. For the purpose of this study, the category chosen for analysis was “nutrition and dietetics”. Web of Science is easy to use and has a simple and advanced search tools. Results of advanced search analysis can be copied and transferred to Microsoft excel for analysis at any point during analysis. Detailed citation analysis of the results of the advanced search can also be easily obtained.

In this study, WoS was used rather than PubMed database. A comprehensive analysis of the advantages and disadvantages of various databases including WoS, PubMed, and Scopus was presented by Falagas et al (2008). In contrast to PubMed, WoS covers most scientific publication and not only the medical and biomedical publication as in the case of PubMed. Furthermore, WoS covers the oldest publications and its records go back to 1900. A major disadvantage of PubMed is the fact that it does not provide citation analysis and therefore does not allow for qualitative analysis of published literature (Falagas et al. 2008). Furthermore, although nutrition is a medical subject, research about nutrition could have been carried out and published in non-medical journals which make PubMed less suitable for this purpose than WoS. In this study, we used ISI WoS rather than Scopus for two main reasons. First of all, no category research is available through Scopus. In contrast, the ISI WoS has a special function in the advanced search that allows investigator to screen for all published articles under the category “nutrition and dietetics”. This is a major advantage of ISI WoS versus Scopus when doing bibliometric analysis of published articles in a special category or field. Second, it is important to note that ISI WoS includes highly citable and influential journals. Such published articles are the ones that affect the practice of nutritional science in the world.

In this study, 21 Arab countries were entered into advanced search engine in WoS followed by “nutrition and dietetics” as WoS category. Palestine was excluded from search keys because the WoS database does list Palestine as an independent state. The results were refined and limited to original research articles and review articles. The time frame for the result was up to year 2012.

### Indices of research productivity

Data were presented as rank order using the standard competition ranking (SCR). Only the 10 top ranked were taken into consideration. The *h*-index for data was also presented. The *h*-index is a country's number of articles (*h*) that have received at least *h* citations (Hirsch 2005). Research activity was adjusted for Arab countries based on population size retrieved from the online databases of the World Bank (World Bank Group 2013). Scientific output was evaluated based on a methodology developed and used in other bibliometric studies (Sweileh et al. 2014b; Zyoud et al. 2014a; Zyoud et al. 2014b; Zyoud et al. 2014c). The collected data were used to generate the following information: (a) total and trends of contributions to research about nutrition and dietetics “during all previous years up to the set date of data analysis (December 31, 2012); (b) Arab countries research productivity and collaboration patterns; (c) journals in which Arab world researchers published; and (d) the citations received by the publications.

## Results

The total number of documents retrieved for worldwide research activity was 195,011. This number represents the global research productivity in the category of Nutrition & Dietetics up to year 2012. The worldwide annual research productivity in nutrition and dietetics showed a significant increase after mid 1970s and reached a maximum annual output of 10,000 published documents in 2012. The leading countries in nutrition and dietetics research were United States of America (USA) (62732; 32.17%) followed by England (13801; 7.08%) and Japan (10579; 5.43%). Worldwide, Egypt and Kingdom of Saudi Arabia (KSA) ranked 38^th^ and 53^rd^ respectively. More than 94% of worldwide nutrition and dietetics documents were written in English and less than 6% were written by 12 other languages mainly French and German languages. Approximately one forth of worldwide published nutrition and dietetics documents were in the research areas of food science/technology and chemistry. Table 1 shows top 10 research areas of worldwide published nutrition and dietetics. Approximately 10% of worldwide research in nutrition and dietetics was published in *Journal of Nutrition*. Table 2 shows the top 10 journals in which worldwide nutrition and dietetics research documents were published. The most productive institutions of nutrition and dietetics research were “Institut National de la Recherche Agronomique (INRA)” in France; Harvard University and University of Minnesota in United States of America with a total of 2429, 2367, 2029 documents respectively.

**Table 1:** **Research areas of worldwide published documents in nutrition and dietetics category**

SCR	Research area	Number of documents N = 195,011 (%)
**1** ^**st**^	Food Science Technology	32472 (16.65)
**2** ^**nd**^	Chemistry	19871 (10.19)
**3** ^**rd**^	Endocrinology Metabolism	15887 (8.15)
**4** ^**th**^	Biochemistry Molecular Biology	10657 (5.47)
**5** ^**th**^	Pediatrics	6354 (3.26)
**6** ^**th**^	Agriculture	6167 (3.16)
**7** ^**th**^	Gastroenterology Hepatology	5891 (3.02)
**8** ^**th**^	Pharmacology Pharmacy	3353 (1.72)
**9** ^**th**^	Psychology	2902 (1.49)
**10** ^**th**^	Psychiatry	2871 (1.47)

*Abbreviations:* SCR = Standard Competition Ranking.

**Table 2:** **Top 10 journals of worldwide published documents in nutrition and dietetics category**

SCR	***Journal***	Number of documents N = (%)
**1** ^**st**^	*Journal of Nutrition*	19054 (9.77)
**2** ^**nd**^	*American Journal of Clinical Nutrition*	15130 (7.76)
**3** ^**rd**^	*Food Chemistry*	13151 (6.74)
**4** ^**th**^	*British Journal of Nutrition*	9138 (4.69)
**5** ^**th**^	*Lipids*	6769 (3.47)
**6** ^**th**^	*Journal of the American Dietetic Association*	6555 (3.36)
**7** ^**th**^	*International Journal of Obesity*	5855 (3.00)
**8** ^**th**^	*Nutrition Reviews*	5802 (2.98)
**9** ^**th**^	*Journal of Pediatric Gastroenterology and Nutrition*	5795 (2.97)
**10** ^**th**^	*European Journal of Clinical Nutrition*	4443 (2.28)

A total of 2062 documents were retrieved for Arab countries which represents 1.0% of the worldwide research productivity. Research activity in nutrition and dietetics from Arab countries remained low and fluctuating all through years until year 2005 and showed a significant increase after that and reached a maximum annual output of 160 published documents in 2012. When retrieved data was analyzed by country, Egypt (742; 35.98%) had the highest quantity of documents followed by KSA (250; 12.12%) and Tunisia (238; 11.54%). Approximately 60% of nutrition and dietetics documents from Arab countries came from three Arab countries, particularly Egypt, KSA, and Tunisia (Table 3). Standardization of research activity using number of population showed that Kuwait (48.31 documents per million inhabitants) was top productive Arab country followed distantly by Lebanon (26.70 documents per million inhabitants) and Bahrain (23.07 documents per one million inhabitants respectively) (Table 3). Of the 2062 documents, 2006 (97.3%) were written in English language while the remaining documents were published in German and French language. Approximately 75% of published nutrition and dietetics documents from Arab countries were in the research areas of food science/technology and chemistry. Top 10 research areas of nutrition and dietetics research from Arab countries are shown in Table 4. Analysis of research areas showed that nutrition-related health research areas such as endocrinology/metabolism, cardiovascular and oncology constituted less than 9% of nutrition and dietetics research activity. A total of 329 (15.96%) nutrition-related diabetes or obesity or cancer documents were published from Arab countries. Noticeable increase in nutrition-related research in these three fields was observed only recently, particularly after year 2008. Countries whose researchers made the most collaboration with investigators in the Arab world include the United States of America (USA); (196; 9.51%) followed by France (188; 9.12%) and England (91; 4.41%).

**Table 3:** **Contribution of each Arab country to 2062 published nutrition and dietetics documents**

Country	Number of documents N = 2062 (%)^a^	Number of documents per million inhabitants
Egypt	742 (35.98)	9.19
KSA	250 (12.12)	8.83
Tunisia	238 (11.54)	22.07
Kuwait	157 (7.61)	48.31
Sudan	126 (6.11)	3.38
Morocco	122 (5.92)	3.75
Lebanon	118 (5.72)	26.70
UAE	85 (4.12)	9.24
Jordan	78 (3.78)	12.38
Algeria	69 (3.35)	1.79
Bahrain	30 (1.46)	23.07
Iraq	29 (1.41)	0.89
Oman	28 (1.36)	8.48
Qatar	22 (1.07)	10.7
Syria	17 (0.82)	0.76
Libya	16 (0.78)	2.58
Yemen	17 (0.82)	0.71
Mauritania	5 (0.24)	1.32

^a^total exceeds 100% because of overlap in some documents among more than one Arab country.

**Table 4:** **Research areas of published documents from Arab countries in nutrition and dietetics category**

SCR	Research area	Number of documents N = 2062 (%)
**1** ^**st**^	Food Science Technology	908 (44.04)
**2** ^**nd**^	Chemistry	629 (30.50)
**3** ^**rd**^	Endocrinology Metabolism	132 (6.40)
**4** ^**th**^	Plant Sciences	62 (3.01)
**5** ^**th**^	Pediatrics	58 (2.81)
**6** ^**th**^	Gastroenterology Hepatology	52 (2.52)
**7** ^**th**^	Pharmacology Pharmacy	49 (2.38)
**8** ^**th**^	Public Environmental Occupational Health	42 (2.04)
**9** ^**th**^	Biochemistry Molecular Biology	32 (1.55)
**10** ^**th**^	Agriculture	25 (1.21)
**10** ^**th**^	Developmental Biology	25 (1.21)
**10** ^**th**^	Oncology	25 (1.21)
**10** ^**th**^	Reproductive Biology	25 (1.21)
**10** ^**th**^	Zoology	25 (1.21)

*Abbreviations:* SCR = Standard Competition Ranking.

Table 5 lists top 10 journals in which nutrition and dietetics research documents from Arab countries were published. Approximately 27% of documents in nutrition and dietetics category published from Arab countries were published in *Food Chemistry* Journal. The most productive institution in Arab country was National research Center in Cairo (212; 10.28%) followed by King Saud University (145; 7.03%) and University of Alexandria (132; 6.4%).

**Table 5:** **Top 10 journals of published documents from Arab countries in nutrition and dietetics category**

SCR	***Journal***	Number of documents N = (%)
**1** ^**st**^	*Food Chemistry*	564 (27.35)
**2** ^**nd**^	*Zeitschrift fur Ernahrungswissenschaft (European Journal of Nutrition)*	105 (5.09)
**3** ^**rd**^	*International Journal of Food Sciences and Nutrition*	88 (4.27)
**4** ^**th**^	*Ecology of Food and Nutrition*	86 (4.17)
**5** ^**th**^	*Nutrition Reports International*	79 (3.83)
**6** ^**th**^	*Nutrition Research*	73 (3.54)
**7** ^**th**^	*Plant Foods for Human Nutrition*	62 (3.01)
**8** ^**th**^	*Nutrition*	61 (2.96)
**9** ^**th**^	*Annals of Nutrition and Metabolism*	60 (2.91)
**10** ^**th**^	*Acta Alimentaria*	59 (2.86)

*Abbreviations:* SCR = Standard Competition Ranking.

Table 6 shows the citation analysis of documents from Arab and neighboring non-Arab countries in nutrition and dietetics category. The total citations for documents published from the Arab countries, at the time of data analysis (June 28^th^, 2014), was higher than that for Israel, or Turkey or Iran. However, when comparison was made at country level, Israel and Turkey published more documents than Egypt or KSA or Tunisia when considered separately. The average citation per documents was lower for those published from Arab countries when considered as a group or when considered separately than those published from Israel or Turkey. Finally, the h-index for documents from Arab countries was lower than that from Israel or Turkey but higher than that from Iran.

**Table 6:** **Research output in nutrition and dietetics from Arab countries compared with that from Turkey, Iran and Israel**

Data/Country	Arab countries	Egypt	KSA	Tunisia	Turkey	Israel	Iran
**Number of population in millions**	400	90	30	11	60	8	80
**Number of published research articles and review articles**	2062	742	250	238	1311	1348	664
**Total number of citations**	22885	7218	2879	3575	21274	29175	9782
**Average citation per document**	11.1	9.5	10.4	13.34	16.23	21.64	14.73
***h*** **-index**	54	37	24	30	57	73	46

## Discussion

Nutrition research is important for human development as well as for improving national health status. No doubt that research regarding nutrition and dietetics is highly needed in Arab countries in order to have baseline data to be used for implementation of nutrition programs directed to solve nutrition-related issues. In our study, a total of 2062 original research articles and reviews related to nutrition and dietetics were retrieved from Arab countries. The actual number of documents might be higher than this given that some journals in which Arab researchers have published about nutrition were not indexed in WoS. For example, Eastern Mediterranean Health Journal is one of the regional journals in the field of public health in which many Arab researchers had published in the field of nutrition; however, documents published in this journal were not counted because it is not indexed in ISI Web of Science. Despite this limitation, our study does give a clear picture and a very close approximation of the quantity, quality, citation analysis, and international collaboration activity of documents in the field of nutrition and dietetics published from Arab countries.

Our results indicated that Kuwait ranked first in research productivity regarding nutrition research measured per million inhabitants. Nutrition-related problems in Kuwait and other gulf countries are due to nutritional transition and overconsumption of food (Musaiger 2011; Hubail and Culligan 2012). A study in seven Arab countries found that, Kuwaiti adolescents had the highest prevalence of obesity (Musaiger et al. 2012b). The findings that Bahrain ranked third in number of documents per million was also expected since the prevalence rates for obesity and diabetes mellitus in Bahrain are higher than the global average (Hubail and Culligan 2012). Obesity and diabetes mellitus have reached alarming limits in Arab Gulf countries and no wonder that researchers from these countries had high research activity regarding nutrition. Furthermore, the relatively good economy of Arab gulf countries participated in pushing medical research activity forward.

Our results showed that interest of Arab researchers in nutrition research showed a dramatic increase only in the last decade. This might be due to alarming epidemiological studies published in late 1990s regarding diabetes mellitus, hypertension and chronic kidney diseases which might be linked to nutrition (El Mugamer et al. 1995; Ibrahim et al. 1995; Musaiger and Al-Roomi 1997; Shaheen and Al-Khader 2005). Arab countries have surpassed Israel, Turkey and Iran in nutrition research activity. However, the quality, as measured by citation analysis, of research from Arab countries was not higher than that produced by Israel or Turkey. This means that although attention to nutrition research is growing, the type and quality of publications is not as those from Israel or Turkey.

Research areas of published documents in the field of nutrition and dietetics from Arab countries were mainly in “Food Science/Technology and Chemistry”. Similar top research areas were found with worldwide published documents in the field of nutrition and dietetics. However, these two research areas constituted approximately 75% of the research area of published nutrition documents from Arab countries compared with approximately 27% for these two research areas for worldwide published nutrition documents. This difference is mainly due to lack of information on chemical composition of local foods and herbs commonly consumed and used in Arab countries. Furthermore, no doubt that processed food technology is new to Arab countries and therefore research in this area is appealing to many of those who are in the field of nutrition, public health, toxicology and industry.

Our study showed that the number of nutrition documents related to diabetes mellitus, or cancer or obesity published from Arab countries was 16% compared with 21% for worldwide nutrition-related diabetes mellitus, pr cancer or obesity. Despite the fact that prevalence rates of diabetes mellitus and obesity have reached alarming limits in some Arab countries, nutrition- related research in these health topics was lagging behind in Arab countries. Recent studies indicated that non-communicable diseases have largely replaced infectious diseases in most Arab countries as a leading cause of death (Mokdad et al. 2014; Rahim et al. 2014). The increased burden of non-communicable diseases in Arab countries could be attributed to several factors. No doubt that modernization, tobacco smoking, health awareness, economic and financial pressuring factors, stress of the unstable and violent political conditions, and inadequate physical activity are among other important contributing factors to the rise of non-communicable diseases in Arab countries (Alwan 1994; Akl et al. 2011; Bull and Dvorak 2013; Maziak et al. 2013; Mokdad et al. 2014). However, nutritional factors and transition in nutritional habits maybe the major contributing factor for the rise in non-communicable diseases in Arab countries. Recently published review articles by Musaiger et al. have described the contribution of current nutritional problems among Arabs and its paramount importance and link to several common non-communicable diseases in Arab countries (Musaiger 2011; Musaiger and Al-Hazzaa 2012). The authors concluded that intervention programs to prevent non-communicable diseases are urgently needed. Such programs need to focus on promoting healthy diet and healthy eating practices in addition to promoting changes in life style pertaining to physical activity (Musaiger and Al-Hazzaa 2012; Musaiger et al. 2013). Similar studies linking nutritional habits and patterns with the rise in non-communicable diseases in Arab countries have been published and demanded urgent national monitoring of factors pertaining to troubling trends of non-communicable diseases in Arab communities (Ng et al. 2011; Musaiger et al. 2012a). Other published article have described the role of nutrition and Arab genetics in emergence of chronic diseases in Middle East and North Africa region (Fahed et al. 2012).

The results of our study showed that journals in the field of chemistry, food science and general nutrition have a good share from nutrition and dietetics publications from Arab countries. It is noteworthy that top 10 journals for publications in nutrition and dietetics from Arab countries did not include any journal in the field of clinical nutrition. In contrast, the worldwide list of top 10 journals for nutrition and dietetics included two clinical nutrition journals, particularly, the *American Journal of Clinical Nutrition* and the *European Journal of Clinical Nutrition*. The difference in the top 10 list journals between Arabs and worldwide reflects differences in research areas and research interest. It was obvious that health and clinically – related journals do not have big share of publications from Arab countries.

To the best of author’s knowledge, this is the first bibliometric study in the field of nutrition and dietetics from Arab countries and does establish a baseline data for future analysis and comparison. Our study is not without limitations, most of which are the same as those of studies performed in other biomedical fields (Sweileh et al. 2014a; Sweileh et al. 2014c,b). First of all, our study is limited by the fact that articles published in journals outside the WoS database were not included. Another limitation is that some publications from Palestine may be affiliated with Israel. Therefore, research activities from Israel were actually from Palestine.

## Conclusion

Our study showed that interest in nutrition and dietetics research is relatively recent in Arab countries. Focus of nutrition research in Arab countries is mainly toward food technology and chemistry with lesser activity toward nutrition-related health research. Arab researchers, particularly in countries with high prevalence rates of obesity, diabetes mellitus, and cancer need to invest in more nutrition research in these fields. International cooperation in nutrition research will definitely help Arab researchers in implementing nutrition research that will lead to better national policies regarding nutrition.

## Authors’ original submitted files for images

Below are the links to the authors’ original submitted files for images. Authors’ original file for figure 1 Authors’ original file for figure 2 Authors’ original file for figure 3

## Abbreviations

EMR: Eastern Mediterranean Region
ISI: Institute for Scientific Information
WoS: Web of Science
KSA: Kingdom of Saudi Arabia
UAE: United Arab Emirates
USA: United States of America
JCR: Journal citation report
SCR: Standard competition ranking
IFs: Impact factors.
