# Supplementary figures and images for: Bibliometric analysis of nutrition and dietetics research activity in Arab countries using ISI Web of Science database
Source: Springerplus. 2014 Dec 10;3:718. doi: 10.1186/2193-1801-3-718 (PMC4320167; doi:10.1186/2193-1801-3-718)

#
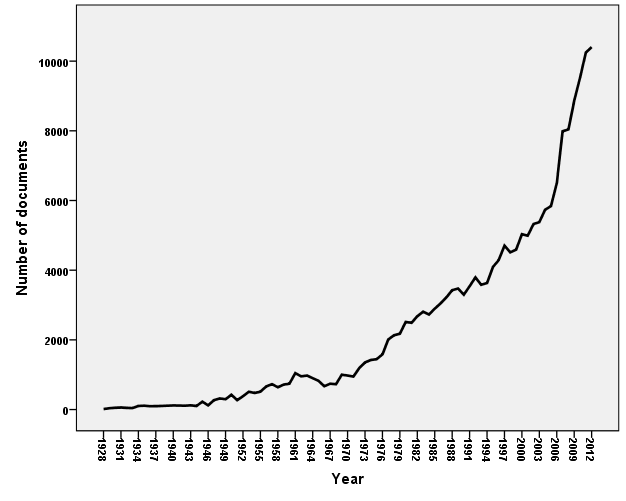


**Figure 1**

Supplement: Supplementary file 1 — Authors’ original file for figure 1 [file 40064_2014_1484_MOESM1_ESM.docx]

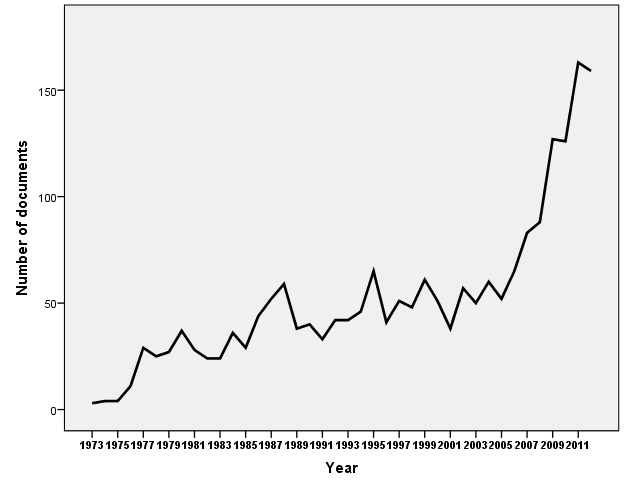


**Figure 2**

Supplement: Supplementary file 2 — Authors’ original file for figure 2 [file 40064_2014_1484_MOESM2_ESM.docx]

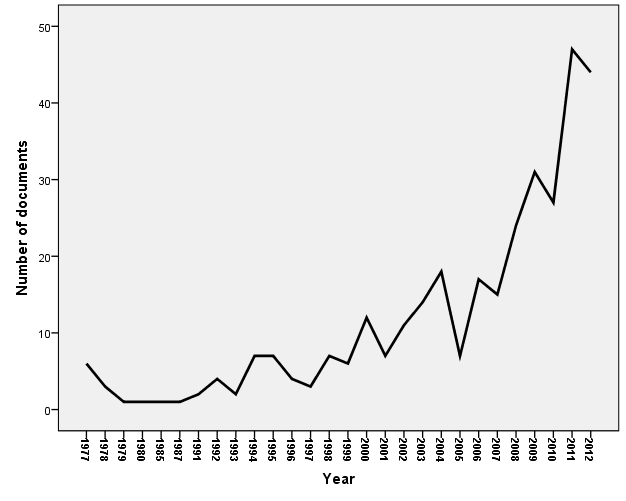


**Figure 3**

Supplement: Supplementary file 3 — Authors’ original file for figure 3 [file 40064_2014_1484_MOESM3_ESM.docx]
